# Alterations in Circulating Progenitor Cell Composition in Rheumatoid Arthritis

**DOI:** 10.3390/cells15080726

**Published:** 2026-04-19

**Authors:** Eva Camarillo-Retamosa, Jan Devan, Camino Calvo-Cebrián, Alexandra Khmelevskaya, Kristina Bürki, Raphael Micheroli, Adrian Ciurea, Stefan Dudli, Caroline Ospelt

**Affiliations:** 1 Center of Experimental Rheumatology, Department of Rheumatology, University Hospital Zurich, University of Zurich, 8091 Zurich, Switzerland; eva.camarilloretamosa@usz.ch (E.C.-R.);; 2 Department of Physical Medicine and Rheumatology, Balgrist University Hospital, Balgrist Campus, University of Zurich, 8008 Zurich, Switzerland

**Keywords:** rheumatoid arthritis, progenitor cells, high-dimensional spectral flow

## Abstract

**Highlights:**

**What are the main findings?**

- CD31 expression is decreased on RA blood cells.
- CD235a+ cells appear more frequently in the peripheral blood of RA patients.

**What are the implications of the main findings?**

- Low CD31 levels might contribute to immune activation in RA.
- Bone marrow activation in RA might lead to increased erythroid progenitors in the peripheral blood.

**Abstract:**

Rheumatoid arthritis (RA) is a chronic autoimmune disease characterised by persistent joint inflammation and systemic immune dysregulation. While bone marrow activation has been linked to RA pathogenesis, direct access to bone marrow tissue for progenitor analysis remains limited by ethical and technical constraints. Analysis of progenitor cells in peripheral blood can serve as a surrogate reflecting bone marrow activation. In this study, we analysed peripheral blood cells from 12 RA patients and 9 healthy controls using high-dimensional spectral flow cytometry with a nine-marker panel (CD45, CD31, CD235, CD133, CD34, CD105, CD271, CD90, PDPN). Flow Self-Organizing Map (FlowSOM) clustering identified 20 distinct cell populations. Additionally, a complementary flow cytometry panel was used to assess CD31 expression on immune subsets in peripheral mononuclear cells (PBMCs) from 9 RA and 9 healthy donors of this cohort. RA patients showed increased CD45^+^CD31^−^ immune cells, but not their putative progenitors. Conversely, putative CD45^+^CD31^int^ progenitors and CD45^+^CD31^int^ mature cells were reduced, along with CD31 expression on T cells. Levels of CD235a^+^ putative erythroid precursors and CD45^+^CD31^+^ progenitors were significantly increased in RA patients. Three putative stromal cell populations were detected in circulation. Together, these findings reveal expanded erythroid precursor populations and reduced CD31 expression on T cells in RA. Our data underscore broad systemic alterations in cellular homeostasis in RA patients. In conclusion, our results suggest that the loss of CD31 expression on immune cell precursors plays a role in age-associated immune remodelling and immune activation in RA and provides the rationale for further studies on erythroblast differentiation and the functional role of erythroblasts in chronic inflammation.

## 1. Introduction

The bone marrow is the primary lymphoid organ in the body with intricate structural and functional determinants that define cell behaviour and fate. All immune cells in our body are initially derived from bone marrow via a differentiation process from immature hematopoietic progenitor cells (HPC). Bone marrow contains an extensive network of ancillary cells supporting haematopoiesis, including fibroblasts, endothelial cells, pericytes and their respective progenitors that, alongside vascular endothelium and mature immune cells, form the bone marrow niche [1,2]. These niches regulate steady-state haematopoiesis and support an increase in haematopoiesis in situations with increased demand, such as infections or severe blood loss [3]. As such, bone marrow cells respond to inflammatory signals and link peripheral inflammatory stress factors to hematopoietic responses of the bone marrow [4,5]. Recent work has demonstrated that inflammatory conditions can fundamentally reprogram haematopoiesis [6]. Chronic exposure to inflammatory cytokines promotes “emergency haematopoiesis,” characterised by altered progenitor differentiation, myeloid bias, reduced regenerative capacity, and persistent immune activation [7]. Studies of the bone marrow niche have further shown that vascular and perivascular compartments actively integrate systemic inflammatory signals, thereby reshaping hematopoietic output [8]. In parallel, inflammatory stress can imprint long-term functional changes on HSC, altering lineage trajectories and sustaining immune dysregulation.

Beyond classical hematopoietic progenitors, emerging evidence suggests that immature cell populations may directly contribute to immune regulation [9]. Erythroid-lineage progenitors—typically confined to the bone marrow—have recently been recognised as active modulators of immune responses under conditions of stress and inflammation. These cells can interact with innate and adaptive immune compartments and influence inflammatory signalling, challenging the traditional view of erythropoiesis as a process solely dedicated to red blood cell production. The detection of erythroid progenitors in peripheral blood, often identified by CD235a (glycophorin A) expression, is therefore increasingly interpreted as a hallmark of systemic hematopoietic stress and inflammation-driven progenitor mobilization [10].

Rheumatoid arthritis (RA) is a chronic, destructive, autoimmune joint inflammation. It affects women more frequently than men and occurs primarily in people of middle age. Inflammation of the synovial tissue in the small joints of the hands and feet is the main clinical feature of RA, but patients are also affected by chronic systemic inflammation, including fatigue, increased cardiovascular risk, and extra-articular symptoms such as skin involvement and serositis [11]. Bone marrow activation has repeatedly been implicated in the pathogenesis of RA, and bone marrow changes observed at the early stages of the disease were shown to be predictive of the development of joint erosions [12,13]. Indeed, evidence from animal models suggests that bone marrow activation precedes the development of joint inflammation [14,15]. In turn, chronic inflammation disrupts bone marrow homeostasis, substantially impacting the function of HPCs, mesenchymal stem cells (MSCs), and their progeny [4], which was also shown in experimental arthritis in mice [16,17].

Despite these strong indications that bone marrow activation contributes to the pathogenesis of RA, direct characterisation of bone marrow progenitor populations in patients remains limited by ethical and technical constraints. As a result, how chronic inflammation reshapes hematopoietic, erythroid, and stromal progenitor output at a systemic level in RA remains poorly understood. We hypothesised that bone marrow activation in RA alters the composition of circulating progenitor and stromal cell populations and therefore aimed to comprehensively map hematopoietic and stromal progenitor cells in the peripheral blood of RA patients using high-dimensional spectral flow cytometry as a surrogate readout of bone marrow activity. By combining multi-marker immunophenotyping with unsupervised clustering approaches, we sought to identify novel alterations in progenitor composition and immune-regulatory cell states that may reflect inflammation-driven remodelling of haematopoiesis in RA.

## 2. Materials and Methods

### 2.1. Patient Recruitment and Cell Isolation

We collected peripheral blood samples from patients diagnosed with RA and HC at the Department of Rheumatology, University Hospital Zurich and Balgrist University Clinic. The demographics and clinical characteristics of the included subjects are depicted in Table 1. RA patients were significantly older than the HC group. All patients diagnosed as having RA fulfilled the 2010 classification criteria [18]. All patients were treated with conventional (c) and/or biologic (b) disease-modifying anti-rheumatic drugs (DMARDs). Complete blood counts were done for all RA patients and were in the normal range (Table 2).

A total of 10 mL of peripheral blood was collected in ethylenediaminetetraacetic acid (EDTA) tubes and centrifuged at 1500 rpm for 10 min at room temperature (RT). The obtained plasma was aliquoted and stored at −80 °C for further analysis, while the red cell pellet was lysed in 1x Red Blood Cell Lysis buffer at RT for over ten minutes according to the manufacturer’s instructions (Biolegend, San Diego, CA, USA). The clear content was centrifuged at 1500 rpm for 10 min at RT. The pellet containing the cells was resuspended in PBS 1X buffer for automated cell counting (Countess 3, Thermo Fisher Scientific, Waltham, MA, USA) with 0.4% trypan blue solution (Invitrogen, Carlsbad, CA, USA). Single-cell suspensions were used for experiments, and aliquots were stored at −80 °C or in liquid nitrogen.

For the cohort analysed by single-cell RNA sequencing, patient recruitment and cell isolation were previously described [19]. Patient’s characteristics for the synovial tissue cohort are described in detail here [19]. Patient’s characteristics for the peripheral mononuclear cell (PBMC) cohort are shown in Table 3.

### 2.2. Blood Phenotype of Progenitor and Rare Cell Populations Using Spectral Flow Cytometry

A single-cell suspension of peripheral blood free of erythrocytes was stained with the cell surface marker panel (Table 4) created on the Easy Panel application from the flow cytometry facility, University of Zurich. Approximately one million cells per donor/patient were stained for viability dye (Zombie NIR) diluted in PBS 1× for 30 min at RT, and after a wash, indirect staining for PDPN was performed by adding the purified antibody diluted in FACS Buffer (PBS 1× with FCS 2% and EDTA 0.5–5 mM) incubated over 30 min at 4 °C protected from light. The secondary antibody conjugated with Viogreen fluorophore was added after a wash with PBS 1×. Upon 30 min of incubation (4 °C, protected from light), antibodies against CD31, CD45, CD235a, CD133 (stem cell biomarker), CD34, CD105, CD271, and CD90 were added to the samples from a mixture prepared in advance according to Table 4. The antibody mixture was incubated for 30 min (4 °C, protected from light) before washing and fixing the stained cells with paraformaldehyde 4% (Thermo Fisher Scientific) for 15 min at RT. The fixative was washed out with PBS 1×. For the acquisition of the spectral cytometer, 5L Aurora (Cytek Biosciences, Fremont, CA, USA) was used at the core facilities at the University of Zurich, where more than 100.000 events were acquired for each tube or well (High-throughput plate loader: flow rate medium 30 µL/min, mix time 4, mix speed 1200 rpm), including FMOs and single staining. The antibody dilutions were optimised in advance in-house using four different dilutions for each CD marker.

### 2.3. Detection of CD31 Expression on Immune Cell Populations

PBMC from nine HC and nine RA donors from the same cohort as for the discovery panel were used. We utilised the highly expressed marker CD45 on immune cells to create unique fluorescent barcodes on each of the samples. Barcoded samples were pooled and co-stained with a previously in-house established broad immunophenotyping panel [20]. Simultaneous staining and analysis of all samples allowed for the analysis to be free of technical bias. The fluorescence barcoding methodology, development of an immunophenotyping panel, gating strategy, and all technical details of the methodological pipeline used for this experiment were described by Devan et al. [20].

### 2.4. Flow Cytometry Data Analysis

The flow cytometry results were analysed using FlowJo^TM^ v10.8 Software (BD Life Sciences, Franklin Lakes, NJ, USA) from the flow cytometry facility at the University of Zurich. Preprocessing included compensation and unmixing using spectral reference files, logicle transformation for fluorescence intensity, and scaling prior to dimensionality reduction. Unmixing files (.fcs) from Aurora 5L were analysed for a supervised gating strategy where the cell population was gated out for debris, droplets, and dead cells to visualize the expression of four cell clusters based on the CD45 and CD31 expression. A gate was created in the plot of CD45/CD31 to ensure that there were no events on the edges. Each sample’s CD45/CD31 quadrant gate was exported to prepare the combined files for the unsupervised analysis using machine learning algorithms from the flowJo (BD Life Sciences) plugins. A unique concatenated file containing 260.000 events from the three groups of combined samples was used for the analysis. Each patient/donor (HC n = 9; RA n = 12) was identified within the corresponding concatenated file. The optimal t-distributed stochastic neighbour embedding (opt-t-SNE) machine learning dimensionality reduction algorithm, the Uniform Manifold Approximation and Projection (UMAP), a dimensionality reduction technique based on triplet constraints, which preserves the global structure of the data, Triplet-based Manifold Approximation (triMAP), and the result of the clustering algorithm Flow Self-Organizing Map (FLowSOM) were all created for the unique concatenated file and assessed for each cohort. Opt-tSNE default algorithm conditions of perplexity (30) and interactions (one thousand) using the Exact KNN algorithm with Barnes-Hunt gradient were created. The UMAP algorithm with default conditions was also applied (Euclidean, fifteen nested neighbours with 0.5 of minimum distance and two components) and triMAP (Euclidean, ten nested neighbours with five outliers). Cell counts and percentages of the parent population were exported into Excel (Windows 10) for statistical analysis in Prism GraphPad v10 (GraphPad, San Diego, CA, USA). To find the proper antibody dilution of the antibody panel, the FlowAI v2.3.2 plugin looked for flow data abnormalities, and the StainIndex v1.8.1 plugin helped to define the negative and positive picks in bimodal populations.

### 2.5. Statistical Analysis

Statistical analysis was performed with GraphPad Prism 11.0.0 and the Shapiro–Wilks test was used to test for normal distribution. The differences between experimental groups were analysed by unpaired t-test when samples followed normal distribution and nonparametric Mann–Whitney tests when not normally distributed. To compare the distribution of cell proportions between the groups, the Chi-square test was performed. Pearson correlation was used for the correlation of cell proportions with age or disease activity. Linear regression with age as a covariate was used if values significantly correlated with age. Data are reported as means with the standard deviation. *p*-values  <  0.05 were considered statistically significant.

## 3. Results

**Figure 1:**
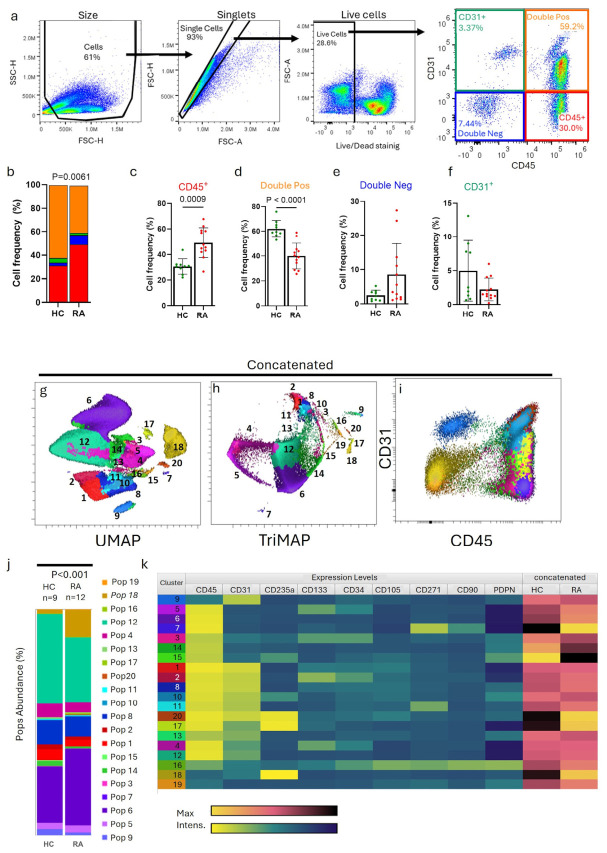
**Peripheral blood cell clusters and their proportions.** (**a**) Cells were gated for size with the forward-light scatter versus granularity/complexity side scatter (FSC vs. SSC) and debris, doublets, and dead cells were excluded. Coloured rectangles highlight four cellular quadrants: CD45^−^CD31^−^ (blue), CD45^+^CD31^−^ (red), CD45^+^CD31^+^ (orange), and CD31^+^CD45^−^ (green). (**b**) Average cell frequency in HC (n = 9) and RA (n = 12). Pairwise statistical Chi-test. Cell frequencies of (**c**) CD45^+^CD31^−^, (**d**) CD45^+^CD31^+^ (**e**) CD45^−^CD31^−^ and (**f**) CD31^+^CD45^−^ cells. Mean and standard deviation (SD); unpaired *t*-test (**c**,**d**) and Mann-Whitney test (**e**,**f**). Unsupervised dimensionality reduction analysis of the measured cell surface proteins in the concatenated file containing healthy controls (HC) (n = 9) and rheumatoid arthritis (RA) (n = 12) with a total of 260.000 life cells is visualised in (**g**) Uniform Manifold Approximation and Projection (UMAP) and (**h**) Triplet-based Manifold Approximation (triMAP) plots. (**i**) Distribution of the populations according to CD45 and CD31 expression. Populations from the Flow Self-Organizing Map (FlowSOM) algorithm are coloured and labelled with numbers from 1 to 20. (**j**) Mean proportions of each coloured and numbered population within the HC (n = 9) and RA (n = 12) cohort. Pairwise statistical Chi-test. (**k**) Heatmap from the FlowSOM cluster analysis shows the expression levels of the markers with intensity levels for each coloured and name-coded cell population (green/yellow panel) and the intensity of each population for the HC (n = 9) and RA (n = 12) (orange/yellow).

We first assessed the distribution of CD45^+^, CD31^+^, double-positive, or double-negative cells. The expression of the CD45 antigen appears at one of the earliest stages of hematopoietic development. It is expressed by all hematopoietic lineage cells except for erythrocytes and mature plasma cells [1]. CD31, the platelet endothelial cell adhesion molecule-1 (PECAM-1), is highly expressed in endothelial cells but is to a lesser extent also found in a variety of other cell types, such as monocytes and lymphocytes, and was shown to suppress antigen receptor signalling in lymphocytes [21]. The co-expression of both markers has been reported in cells from the vascular and hematopoietic systems [22,23]. We considered CD45^−^CD31^−^ double-negative cells as putative stromal cells. Flow cytometry analysis for CD45 and CD31 expression showed four well-defined cellular compartments (Figure 1a). Peripheral blood contained, on average, 30% of immune cells (CD45^+^), 7% of putative stromal cells (CD45^−^CD31^−^), 3% of endothelial cells (CD45^−^CD31^+^), and 60% of cells expressing both CD45 and CD31.

Patients with RA showed a significantly different pattern of cell frequencies (*p*-value = 0.0061) when compared to the HC cohort (Figure 1b). RA samples presented with a significantly increased percentage of circulating immune cells expressing CD45 (49 ± 11%, *p*-value = 0.0009) when compared to HC (31 ± 6%) (Figure 1c), reflecting the activation of the immune system in RA patients. Moreover, RA patients had significantly reduced proportions of the large CD45^+^/CD31^+^ compartment (40 ± 10%, *p*-value < 0.0001) (Figure 1d). Some samples from the RA cohort had an increased percentage of double-negative stromal cells (Figure 1e). In contrast, the percentage of circulating CD45^−^CD31^+^ cells seemed reduced in RA compared to the HC cohort (Figure 1f); however, statistical significance was not reached.

To further explore these alterations, markers previously used to identify progenitors from the haematopoietic branch, such as CD235a, CD133, and CD34, from activated stages of the endothelium (CD105) and the stromal/skeletal cell populations (CD271, CD90 and PDPN), were multiplexed with CD45 and CD31 for each sample. To assess how these markers behave on the cells and to detect cellular subsets, a self-organising map clustering (FlowSOM) was performed. The FlowSOM clustering algorithm identified twenty different cellular clusters according to the expression of each CD marker at the single cell level (Figure 1k). The selection of 20 FlowSOM clusters was based on empirical testing of multiple cluster numbers (n = 8–25), observed stability around n = 15–20, software defaults, expert consultation, and published recommendations [24,25]. Double negative, double positive, and CD45^+^ cell populations were partitioned into different subpopulations, while CD31 single-positive cells did not further subcluster by using these markers. We visualised these subpopulations in UMAP (Figure 1g) and Triplet-based Manifold Approximation (TriMAP) (Figure 1h) plots, as well as in relation to the CD45/CD31 expression (Figure 1i). While UMAP is widely used for visualising high-dimensional single-cell data due to its ability to preserve local structure, its 2D proximity relationships can be misleading, as global distances between clusters are often distorted. In contrast, TriMAP employs triplet-based constraints to better preserve global structure, resulting in more faithful representations of relative distances between clusters [26]. Mapping of the proportional abundance revealed significant diversity of the identified cellular subclusters between HC and RA (*p*-value < 0.001) (Figure 1j,k).

FlowSOM identified six distinct populations within the CD45^high^ compartment (Pop 3, 5, 6, 7, 14, 15 in Figure 1k). Pop 6 expressed only CD45 and corresponded to the population shown in Figure 1c. The other CD45^+^ populations, Pop 3, 5, 7, 14, and 15, expressed variable levels of CD90 or PDPN or the progenitor markers CD235a, CD271, CD133, and CD34 (Figure 1k). The global distribution TriMAP plot showed Pop 5 and Pop 14 in close proximity to Pop 6 (Figure 1h). Pop 5 expressed CD34 and CD133 (Figure 1k). Both markers define HSCs [27]. Pop 3 was a rare, separated cell population expressing CD34 and CD133 as well as PDPN (Figure 1k). In RA, it was previously shown that circulating progenitor cells expressing CD34 were associated with inflammation, oxidative stress, and arterial stiffness, indicating a potential link between these factors and increased risk for cardiovascular disease [28]. However, we did not observe an increase in these two CD45^+^CD34^+^ cell populations in the peripheral blood in our RA cohort (Supplementary Table S1). Pop 7 defined a small, independent cluster of immune cells expressing CD271, the Nerve Growth Factor Receptor (NGFR), a marker of multipotency [29]. Pop 15 was characterised by the expression of PDPN and low expression of CD235a. Also, these subpopulations did not differ in their frequency between healthy controls and RA samples (Supplementary Table S1). In summary, while we detected an increase in CD45^+^ cells in the blood of patients with RA, we did not find increased levels of CD45^+^ progenitors in the periphery in this cohort.

**Figure 2:**
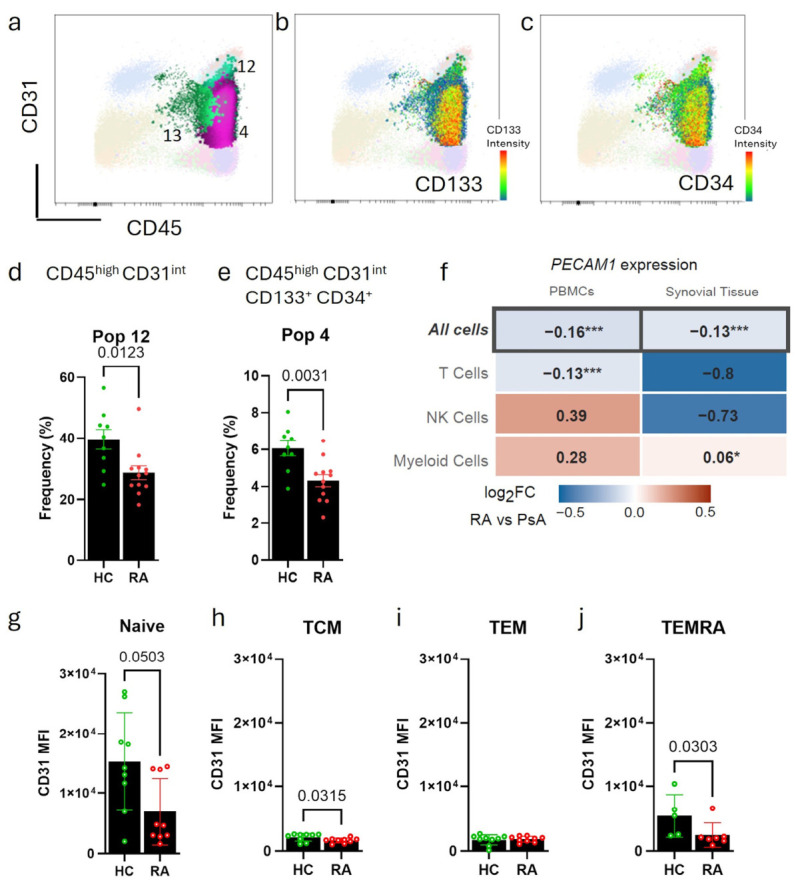
**CD45^+^CD31^int^ cells were reduced in RA patients.** (**a**) CD45/CD31 dot plot showing populations 12, 4 and 13. Pop 4 expressed high levels of (**b**) CD133 and (**c**) CD34 (red dots). Bar graphs show a significantly reduced frequency in the percentage of (**d**) Pop 12 and (**e**) Pop 4 in RA patients (n = 12) when compared to HC (n = 9). Mean and SD; Mann–Whitney and *t*-test, respectively. (**f**) Heat map showing log fold change differences in *PECAM1* expression in peripheral mononuclear cells (PBMCs) (RA n = 5, PsA n = 6) and in immune cells within synovial tissues (RA n = 13, PsA n = 12). MAST test, * *p* < 0.05, *** *p* < 0.001. Mean fluorescence intensity (MFI) of CD31 expression on (**g**) naïve CD4 T cells, (**h**) central memory (TCM), (**i**) effector memory (TEM), and (**j**) T cells expressing CD45RA (TEMRA). n = 9 per group, all Mann–Whitney, except in G, the *t*-test was used. All dot plots show mean and SD.

Within the CD45^+^CD31^+^ compartment, ten subpopulations were identified (Figure 1k). Most of these, and in particular the CD45^high^CD31^high^ populations Pop 1, 2, 8, 10, 11, and 20, were rare (Supplemental Table S1) and, except for Pop 20, clustered closely together in the triMAP (Figure 1h). Pop 1 was reminiscent of the mature CD45^high^CD31^high^ population previously described as erythro-myeloid progenitors (EMP) [30] and showed increased frequency in RA blood samples (2.178 ± 1.4 vs. 2.827 ± 1.50, *p*-value = 0.041) (Supplementary Table S1). Pop 12 (CD45^+^CD31^int^) had a dimmer expression of CD31 (Figure 2a), but a much higher frequency and was in close proximity to the CD45^+^ immune cell population (Pop 6) in the triMAP (Figure 1h). Therefore, this population might reflect immune cells with expression of CD31. This population reduced from 40% in healthy controls to 29% in RA (Figure 2d), probably reflecting the reduction of double-positive cells seen in Figure 1d. The CD45^+^CD31^int^ Pop 4 diverged from Pop 12 and mapped in close proximity to the CD45^+^CD133^+^CD34^+^ HSCs population (Pop 5) (Figure 1h). Similar to the CD45^+^CD31^−^ HSC Pop 5, Pop 4 also expressed high levels of CD133 (Figure 2b) and CD34 (Figure 2c), but also a clear expression of CD31 (Figure 2a). Interestingly, Pop 4 also presented with a reduced frequency in RA (Figure 2e).

CD31 expression on immune cells is known to be affected by age, and it was, for instance, shown that the presence of CD31^+^ T cells decreased in healthy controls with increasing age [31]. Because in our cohort, there was a significant age difference between the RA (62 ± 7 years) and the HC population (47 ± 9 years) (Table 1), we tested the correlation between the proportions of the twenty populations with age. Out of the twenty subpopulations, only the double-positive Pop 4 and Pop 12 and the double-negative Pop 18 significantly correlated with age (Supplementary Figure S1a–c). When we used age as a covariate in a linear regression model, there was still a statistical trend (*p*-value = 0.078) of decreased levels of the progenitor Pop 4 in RA, and age did not appear to have a significant effect on the proportions (*p*-value = 0.466). Pop 12 and Pop 18 were not significantly altered in this statistical model (*p*-value = 0.234 and *p*-value = 0.192, respectively). But also here, the model did not provide statistical evidence that age influenced the outcome (*p*-value = 0.255 and *p*-value = 0.433). However, given the small sample size, the statistical analysis should be viewed with caution. Together, this indicated that although age is a determinant of CD31 expression in immune cells, RA might have an independent effect on the expression of CD31 in the CD45^+^CD31^int^ population. To confirm our findings in an independent cohort, we re-analysed a previously published in-house dataset of single-cell RNA sequencing of age-matched PBMCs and synovial biopsies of RA patients and patients with psoriatic arthritis (PsA) [19]. Compared to patients with PsA, immune cells from RA patients showed significantly less expression of *PECAM1* (encoding for CD31) in T cells in peripheral blood, but not in other immune cells (Figure 2f). Overall, *PECAM1* was also significantly reduced in immune cells in the synovial tissue and most strongly in T cells but did not reach statistical significance. However, without the addition of a canonical lineage marker, we could not define whether these changes are based on the reduction of the putative progenitor Pop 4 or whether they represent a global change in the proportions of mature immune cells (e.g., a decrease in naïve T cells [CD31^+^] and an increase in effector memory T cells [CD31^−^].

Since a regulatory role for CD31 in T cell activation has previously been shown [32], we analysed the expression of CD31 on subsets of CD4 T cells in a subgroup of patients from our original cohort. CD31 expression was lower on naïve (CCR7^+^CD45RA^+^) CD4 T cells in RA compared to healthy donors (Figure 2g), as well as in RA T Central Memory cells (TCM; CCR7^+^CD45RA^-^) (Figure 2h), and Terminally Differentiated Effector Memory T cells (TEMRA; CCR7^-^CD45RA^+^) (Figure 2j), but not in RA T effector memory (TEM, CD45^−^CCR7^−^) cells (Figure 2i). The comparison of CD31 expression on these different CD4 T-cell functional maturation stages showed that naive T cells expressed significantly higher CD31 compared to other functional maturation stages of T-cells, as previously described [23]. Expression of CD31 negatively correlated with age in the naïve T cell population (Supplementary Figure S1d), but not on the more mature T cell stages analysed. After accounting for the influence of age on CD31 expression in the statistical model, CD31 expression was still significantly lower in RA TCM (*p*-value < 0.001), and there was a statistical trend for lower expression of CD31 in RA TEMRA (*p*-value = 0.092) compared to healthy controls.

**Figure 3:**
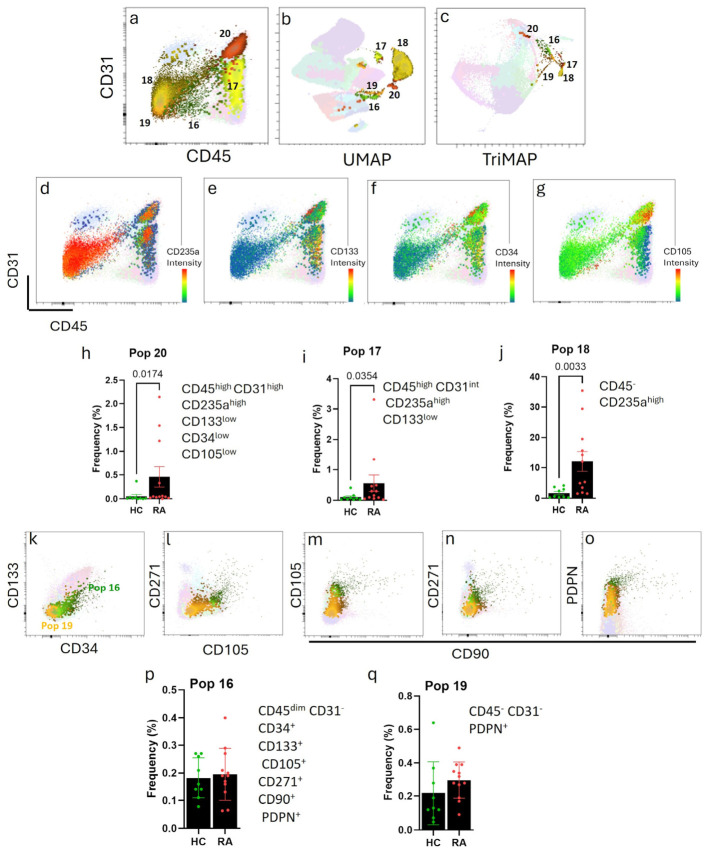
**CD235a^+^ and CD45^−^CD31^−^ stromal cells circulating in peripheral blood.** (**a**) CD45/CD31 dot plot showing populations 16, 17, 18, 19 and 20. (**b**) UMAP shows the size of each cluster, and (**c**) TriMAP shows the relative cluster location. CD45/CD31 plots highlighting the expression of (**d**) CD235a, (**e**) CD133, (**f**) CD34 and (**g**) CD105. The intensity scale indicates high expression of the marker in red. Frequency of (**h**) Pop 20, (**i**) Pop 17, and (**j**) Pop 18. Mann-Whitney test. Dot plots showing the expression of (**k**) CD34/CD133, (**l**) CD271/ CD105, and CD90 with (**m**) CD105, (**n**) CD271, and (**o**) PDPN in Pop 16 and Pop 19. Frequency of (**p**) Pop 16 and (**q**) Pop 19. *t*-test for Pop 16; Mann–Whitney test for Pop 19. All dot plots show mean and SD, n = 12 RA and 9 HC.

The CD45^high^CD31^high^ Pop 20, the CD45^high^CD31^int^ Pop 17, and the double-negative Pop 18 (Figure 1k and Figure 3a) had a remarkably high expression of the erythrocyte marker CD235a (Figure 3d), which we confirmed by a manual gating strategy (Supplementary Figure S2). These populations were separated from the other populations (Figure 3b) and clustered together in the TriMAP (Figure 3c). Although all three populations were statistically more abundant in RA patients compared to controls, only some patients had strongly increased levels of these populations, and in healthy controls and in most RA patients, the frequency of these populations was very low (Figure 3h–j, Supplemental Table S1). Strikingly, however, the double-negative population 18 represented more than 10% of all cells in half of the RA patients (Figure 3j). The high proportion of this cell population in certain patients could not be explained by clinical factors such as CRP, disease activity (DAS28), seropositivity, or treatment.

Based on the expression of the progenitor markers CD133 (Figure 3e) and CD34 (Figure 3f) by Pop 17 and Pop 20 and the additional expression of CD105 by Pop 20 (Figure 3g), we hypothesise that these populations represent different progenitor stages in erythropoiesis. Curiously, Pop 18 did not express any of these progenitor markers. Due to their size and the fact that we thoroughly eliminated mature erythrocytes from our analysis (see Section 2), we can exclude that Pop 18 are mature erythrocytes, but based on the used markers, we cannot determine which stage of erythropoiesis they reflect. As mentioned above, this subpopulation significantly increased with increasing age (Supplementary Figure S1c) and was not statistically significant in a linear regression model with age as a covariate. Nevertheless, together the results show an increased presence of erythroid precursor cells in the peripheral blood of some RA patients, which warrants further investigation in future studies.

In addition to the CD235a^+^ Pop 18, we identified two more double-negative populations, Pop 16 and Pop 19. Pop 16 expressed the progenitor markers CD34, CD133, CD271 and CD105 (Figure 3k,l) as well as CD90 (Figure 3n) and PDPN (Figure 3o). In addition, Pop 16 expressed CD45 very dimly (Figure 1k). Altogether, this suggested that Pop 16 might be an MSC-like population. The frequency of this population was very low (mean percentage < 0.2) and was not different between HC and RA patients (Figure 3p).

Pop 19 showed a clear expression of PDPN (Figure 1k and Figure 3o). The expression of PDPN in the double-negative compartment in PB was previously described in so-called pre-inflammatory mesenchymal (PRIME) CD45^−^CD31^−^ cells associated with flares of RA patients [33]. The percentage of these cells increased from 0.2% in HC to 0.3% in RA patients but did not reach statistical significance (Figure 3q).

## 4. Discussion

In the current work, we used high-dimensional spectral flow cytometry to analyse cell populations in the peripheral blood of patients with RA in comparison to healthy controls. By combining nine different cell markers, we could identify 20 different subpopulations of blood cells. We found decreased percentages of CD45^+^CD31^int^ progenitor and CD45^+^CD31^int^ mature cells, as well as decreased levels of CD31 expression on T cells in RA patients. Furthermore, our results show an increased appearance of putative erythroid precursor populations in the peripheral blood of patients with RA.

Recent high-dimensional atlases have provided a detailed characterisation of human haematopoietic progenitors using single-cell RNA sequencing and mass cytometry [34,35]. The use of spectral flow cytometry in our study enables sensitive detection and initial characterisation of rare circulating progenitor subsets at the protein level. Given the scarcity of datasets addressing circulating progenitor populations in human RA, our data offer a complementary and relevant perspective, expanding current knowledge of haematopoietic dysregulation in RA and providing a basis for future studies.

As expected, most of the cells in the peripheral blood were CD45^+^. Interestingly, the CD45^+^CD31^−^ population increased in RA patients while the CD45^+^CD31^+^ population decreased. This was mainly based on the diminished presence of a large population of CD45^+^CD31^int^ cells in RA patients. Dim CD31 expression can be found in lymphoid and myeloid cells [36], as well as in HCS [37]. CD31 is expressed by naïve B and T cells and is downregulated during differentiation into memory cells [21,38]. Therefore, the decreased presence of CD45^+^CD31^int^ cells in RA might simply reflect the shift from naïve to memory cells, and future studies are needed, incorporating lineage markers and functional assays to clarify these results. However, the close connection between the CD45^+^ HCS Pop 5 and the CD45^+^/CD31^int^ HCS Pop 4 in the TriMAP suggested that Pop 4 is a subpopulation of Pop 5 expressing CD31 and a progenitor population of CD31-expressing lymphoid and myeloid cells. Lack of CD31 expression was shown to promote immunogenic dendritic cell maturation [39]. On T cells, loss of CD31 increases clonal expansion and hampers regulatory functions [40]. Furthermore, CD31 inhibits B-cell and T-cell receptor signalling and thus dampens B and T-cell activation [41,42]. Patients with coronary heart disease were shown to have decreased frequencies of CD31^+^ T regulatory cells and CD31^−^ T regulatory cells expressed less FoxP3 and showed attenuated proliferation and immunosuppressive properties [43]. In RA synovial tissue, a lack of CD31 in the immune synapse could be shown and CD31 agonists ameliorated collagen-induced arthritis [39]. Intriguingly, a decrease of angiogenic T cells (Tang) as well as of CD34^+^VEGFR2^+^CD133^+^ angiogenic progenitor cells (EPC) was shown in patients with RA [44]. Tang are CD3^+^CD31^+^CXCR4^+^ cells that are required for EPC differentiation and thus support endothelial repair [45]. In RA, their decrease correlated with disease activity and antibody positivity. Diminished Tang and EPC in RA could be reflected by our results, and our Pop 12 and Pop 4 might include Tang and EPC, respectively. However, both are very rare populations, and Tang only make up 2–5% of all T cells [44]. Therefore, we think that most of the differences we observed were not based on a reduction of Tang or EPC in RA. To the best of our knowledge, there is no other study that analysed the expression of CD31 in RA peripheral blood immune cells, but it is feasible to assume that the lack of CD45^+^CD31^int^ cells and the diminished CD31 expression on T cells in RA are contributing to immune cell activation. Since our study was compromised by the significant age differences between healthy control subjects and RA patients, and treatment effects could not be ruled out, a thorough analysis of CD31 expression on immune cells in healthy control subjects of the same age and untreated RA patients is necessary to further confirm this interesting observation.

We found three different CD235a^+^ cell populations at higher percentages in RA patients compared to healthy controls (Pop 17, 18, 20). CD235a (glycophorin A) is expressed on erythroblasts and mature erythrocytes. Since we eliminated mature erythrocytes from the blood and gated out the possible remaining ones by size, we assume that the CD235a-expressing cells in our dataset are putative erythroid precursors (nucleated red blood cells). An increase in erythroblasts is observed in several haematological disorders and life-threatening conditions such as sepsis, acute coronary syndrome, or multi-organ failure [46] but has never been described in RA. Their appearance in the peripheral blood might be a consequence of bone marrow activation in RA. Notably, in our RA cohort, haemoglobin, haematocrit, erythrocyte and platelet counts, MCV, MCH, and MCHC were in the normal range. However, we cannot exclude that the appearance of these cells is linked to subclinical hematopoietic stress or an early, compensatory mechanism in response to anaemia in chronic disease. Erythropoiesis can also be induced from CD45^+^CD31^+^ blood cells [22]. Expression of CD235a in this so-called EMP was shown to be induced by VEGF [47]. Therefore, increased differentiation of EMP into erythroblasts might be induced by the inflammatory milieu in RA with increased levels of cytokines such as VEGF. Indeed, the CD45^+^CD31^+^ population (Pop1) was significantly increased in the blood of RA patients. The CD235a^+^ Pop 20 expressed CD45 and CD31 as well as the progenitor markers CD133, CD34, and CD105. Pop 17 remained CD45^+^ but showed only dim expression of CD31 and CD105. Pop18 finally was CD45^+^CD31^−^. This gradual loss of CD31 was previously observed in in vitro differentiated erythroblasts from CD45^+^CD31^+^ blood cells [47] and thus indicates that Pop 20, 17, and 18 might represent increasingly mature stages of erythroblasts derived from EMP. Currently, there is no functional role apart from erythropoiesis described for erythroblasts. Since erythrocytes have been found to play a role in regulating innate immune activation [48], the role of these nucleated erythroid progenitors in the immune system would be interesting to explore.

CD45^−^CD31^−^PDPN^+^ cells in the circulating blood have been previously described as PRIME cells and were shown to be increased in the blood of patients with RA compared to healthy controls [33]. We could differentiate two CD45^−^CD31^−^PDPN^+^ populations in the blood, a CD45^dim^CD31^−^PDPN^+^ population expressing also CD34, CD133, CD105 and CD271 (Pop 16) and a CD45^−^CD31^−^PDPN^+^ population (Pop 19). Due to the marker expression, we assume that the first population represents an MSC-like population in peripheral blood and might be a precursor of the latter. Interestingly, we only saw increased frequencies of the more differentiated Pop 19, but not in the MSC-like population. While this difference was not statistically significant, it showed that CD45^−^CD31^−^PDPN^+^ cells seem to be expanded in some RA patients and indicated that the appearance of PRIME cells in RA patients who flare might not be based on increased progenitor release.

The increase/decrease of the measured cellular phenotypes in RA patients did not correlate with CRP or disease activity. The lack of statistical significance may reflect the limited sample size. However, given the dynamic nature of CRP and disease activity, being elevated during flares and normalised thereafter, the observed changes may indicate chronic or subclinical immune remodelling rather than acute inflammation.

Our study is limited by the number of samples, the lack of a confirmatory cohort, and the fact that patients were not treatment-naive. Our cohort comprises patients with long-standing and intensively treated RA, and we cannot exclude that our findings are not seen in early untreated patients. Unfortunately, our cohort is, in general, not very well defined clinically, and a larger study involving patients in which the reduction in CD31 on T cells or the presence of CD235a^+^ cells in the blood can be correlated with further clinical data, such as disease duration, bone erosion, and patient-reported outcomes, would be desirable. Furthermore, due to the small cohort size, a statistical trend (*p* = 0.062) is only visible for populations 4 and 18 when the Bonferroni correction of the *p*-value is applied (Supplementary Table S1). Moreover, given that progenitor cell clusters are rare, a higher number of cells per patient would be needed to assess the robustness of the changes observed for some of the cell populations. Additionally, full phenotyping of new cellular populations would require more cellular makers, and functional characterisation is lacking. Nevertheless, our data provides interesting insights into the presence of progenitor populations in the peripheral blood and opens new fields of research into the role of erythroblasts and CD31 expression on immune cells in RA.

## 5. Conclusions

In conclusion, our preliminary study opens up two new intriguing areas of research in RA. We provide evidence that CD31 expression on T cells is diminished in RA patients, which might be associated with a loss of CD31-mediated inhibitory signalling in circulating immune cells, consistent with enhanced immune activation. Furthermore, we found that RA patients show increased circulating CD235a^+^ cell populations, which supports the hypothesis that RA is associated with aberrant or stress-induced erythropoiesis, likely reflecting bone marrow activation.

## Figures and Tables

**Table 1:** **Socio-demographics of the studied population.** NA = not assessed, RF = rheumatoid factor, ACPA = anti-citrullinated peptide antibodies, CRP = C-reactive protein, NSAIDs = Nonsteroidal anti-inflammatory drugs, cDMARDs = conventional disease-modifying anti-rheumatic drugs, b/smDMARDs = Biological/small-molecule DMARDs.

	Age	Sex	Smokers	RF + ACPA	DAS28	CRP	NSAID	cDMARD	bDMARD	smDMARD	Corticosteroids in the Last 3 Months
	years					mg/L					
RA1	70	F	no	positive	5.41	30					
RA2	62	F	no	positive	4.5	5.1		sulfasalazine		upadacitinib	
RA3	59	F	no	negative	3.55	6.6	x				
RA4	67	F	yes	positive	4.18	1.9				upadacitinib	x
RA5	77	M	no	negative	2.53	0.6		methothrexate			
RA6	67	F	no	NA	3.54	0.6	x	methothrexate			
RA7	65	F	no	positive	2.1	0.8		hydroxychloroquine, leflunomide	rituximab		
RA8	53	F	no	negative	3.69	0.6				upadacitinib	
RA9	54	F	NA	negative	2.1	1.9				tocilizumab	
RA10	59	F	yes	negative	3.41	0.6			rituximab		
RA11	64	F	no	positive	5.73	1		leflunomide	abatacept		
RA12	52	F	NA	NA	NA	0.5					
HC1	45	F	NA	NA	NA	NA					
HC2	44	F	NA	NA	NA	NA					
HC3	62	F	NA	NA	NA	NA					
HC4	38	F	NA	NA	NA	NA					
HC5	30	F	NA	NA	NA	NA					
HC6	47	F	NA	NA	NA	NA					
HC7	52	F	NA	NA	NA	NA					
HC8	48	F	NA	NA	NA	NA					
HC9	55	M	NA	NA	NA	NA					

**Table 2:** **Blood counts of RA patients.** g/L = gram/litre, l/L = litre/litre, t/L = teralitre/litre, fl = femtolitre, pg = picogram.

	Haemoglobin	Haematocrit	Erythrocytes	Mean Corpuscular Volume	Mean Corpuscular Haemoglobin	Mean Corpuscular Haemoglobin Concentration	Thrombocytes	Leukocytes
	g/L	l/L	t/L	fl	pg	g/L	g/L	g/L
normal range	117–153	0.350–0.460	3.9–5.2	80–100	26–34	310–360	143–400	3.0–9.6
RA1	113	0.344	3.74	92	30.2	328	295	6.86
RA2	120	0.362	3.92	92.3	30.6	331	304	6.98
RA3	135	0.391	4.52	86.5	29.9	345	240	6.16
RA4	156	0.479	5.37	89.2	29.1	326	218	7.33
RA5	133	0.39	4.04	96.5	32.9	341	174	5.13
RA6	131	0.396	4.24	93.4	30.9	331	216	6.05
RA7	137	0.416	4.9	84.9	28	329	259	7.25
RA8	129	0.365	3.93	92.9	32.8	353	213	4.17
RA9	121	0.378	4.27	88.5	28.3	320	226	4.06
RA10	121	0.355	4.1	86.6	29.5	341	392	7.97
RA11	128	0.395	4.24	93.2	30.2	324	148	5.01
RA12	122	0.361	4.06	89	30	338	269	5.08

**Table 3:** Socio-demographics of the studied population for single-cell RNA sequencing.

	Age	Sex	Smokers	RF + ACPA	DAS28	NSAID	cDMARD	Corticosteroids in the Last 3 Months
	years							
RA1	48	M	yes	positive	4.12			x
RA2	60	M	no	positive	5.04	x	methothrexate	x
RA3	44	F	no	positive	2.61		methothrexate, salazopyrine	x
RA4	41	M	yes	positive	6.26			
RA5	64	M	no	positive	5.5		methothrexate	
PsA1	54	F	no	NA	2.6			x
PsA2	31	M	NA	NA	2.8			
PsA3	23	M	no	NA	3.11		methothrexate	
PsA4	48	F	no	NA	3.48		methothrexate	
PsA5	58	M	no	NA	4.35	x	methothrexate	
PsA6	70	F	yes	NA	3.27	x	methothrexate	

**Table 4:** Cytometry panel specifying CD markers and antibody dilutions.

Marker	Fluorophore	Code	Brand	Dilution	Cytek5L Channel
Viability	Zombie NIR	423106	Biolegend	1/3000	R7
PDPN	Purified	130-107-018	Miltenyi Biotec (Bergisch Gladbach, Germany)	1/100	-
Secondary	VioGreen	130-113-859	Miltenyi Biotec	1/50	V8
CD31	BUV805-A	742013	Becton Dickinson (Franklin Lakes, NJ, USA)	1/150	UV16
CD34	PerCP-Vio700-A	130-113-181	Miltenyi Biotec	1/450	B10
CD45	FITC-A	130-113-117	Miltenyi Biotec	1/150	B2
CD90	APC-A	130-114-861	Miltenyi Biotec	1/50	R1
CD105	VioBlue-A	130-099-666	Miltenyi Biotec	1/100	V3
CD133	PE-Vio770-A	130-112-159	Miltenyi Biotec	1/150	YG9
CD235a	BUV395	563810	Becton Dickinson	0.2	UV2
CD271	PE-A	130-113-421	Miltenyi Biotec	1/450	YG1

## Data Availability

The data is publicly available on the Mendeley Data repository: Camarillo, Eva; Devan, Jan; Ospelt, Caroline (2026), “Flow Data”, Mendeley Data, V1, doi: 10.17632/g45kc34nh4.1.

## Supplementary Materials

The following supporting information can be downloaded at: https://www.mdpi.com/article/10.3390/cells15080726/s1. Supplementary Table S1. Cellular Populations frequency and significance. The table contains the cell surface markers expressed for each cell population received from the FlowSOM clustering analysis. The mean and SD for the frequency (percentage) of each cell population within the cohorts, HC (n = 9) and RA (n = 12), are listed with the *p*-value, the statistical significance, and the corrected *p*-value (Bonferroni). Supplementary Figure S1. Relation of age with subpopulations. Pearson correlation analysis between age and (**a**) Pop 4, (**b**) Pop 12 (**c**) Pop 18, and (**d**) naive T cells. Supplementary Figure S2. Manual gating strategy and dot plots of each patient. (**a**) Manual sequential flow cytometry gating strategy. Bivariate dot plot showing CD235a and CD45 expression profiles for (**b**) HC samples and (**c**) RA samples.
