# Supplementary material for: Alterations in Circulating Progenitor Cell Composition in Rheumatoid Arthritis
Source: Cells. 2026 Apr 19;15(8):726. doi: 10.3390/cells15080726 (PMC13114382; doi:10.3390/cells15080726)
Supplement: Supplementary file 1 [file cells-15-00726-s001.zip › cells-4235021-supplementary.pptx]

## Slide 1
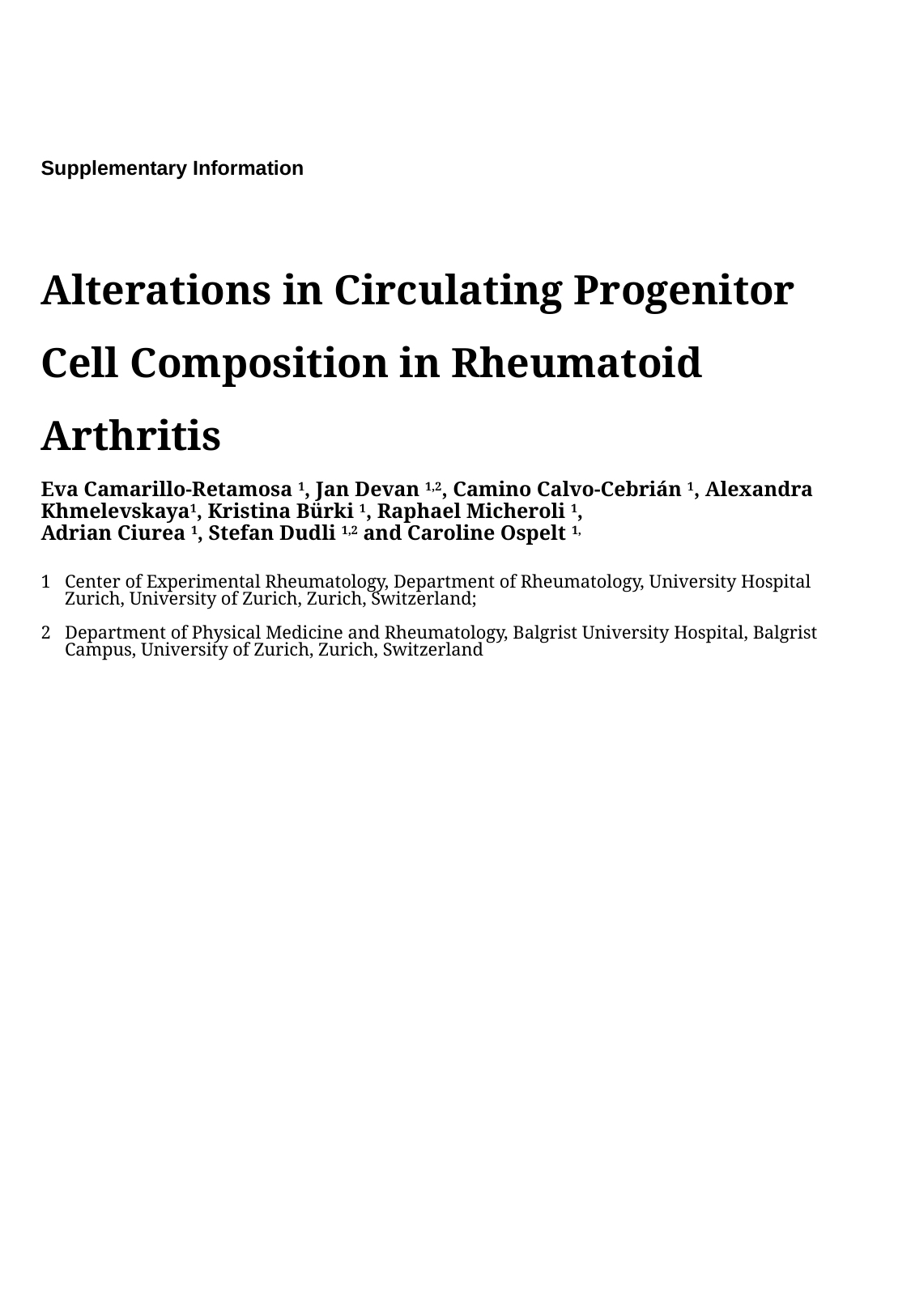

Supplementary Information
Alterations in Circulating Progenitor Cell Composition in Rheumatoid Arthritis
Eva Camarillo-Retamosa 1, Jan Devan 1,2, Camino Calvo-Cebrián 1, Alexandra Khmelevskaya1, Kristina Bürki 1, Raphael Micheroli 1,
Adrian Ciurea 1, Stefan Dudli 1,2 and Caroline Ospelt 1,
Center of Experimental Rheumatology, Department of Rheumatology, University Hospital Zurich, University of Zurich, Zurich, Switzerland;
Department of Physical Medicine and Rheumatology, Balgrist University Hospital, Balgrist Campus, University of Zurich, Zurich, Switzerland

## Slide 2
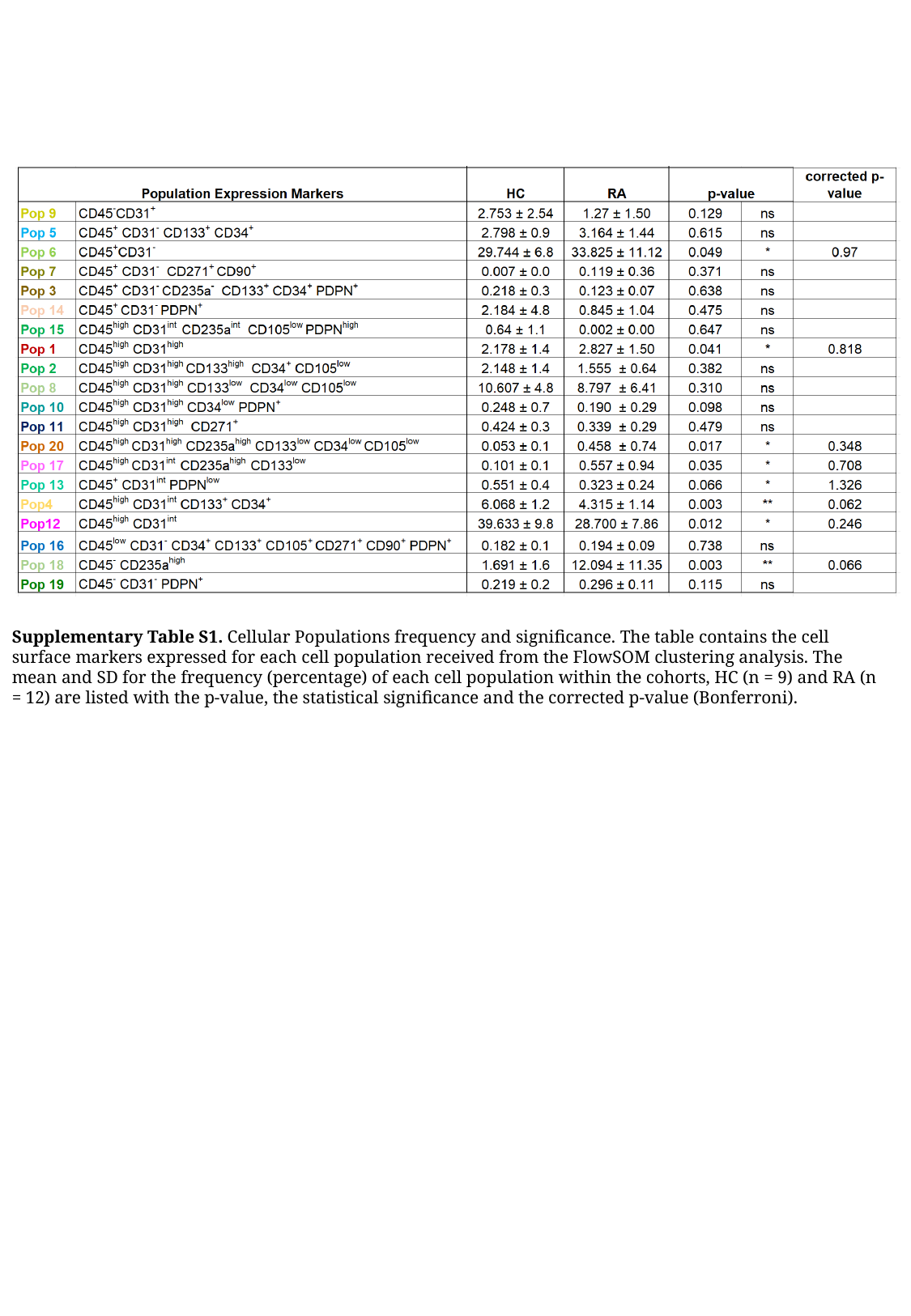

Supplementary Table S1. Cellular Populations frequency and significance. The table contains the cell surface markers expressed for each cell population received from the FlowSOM clustering analysis. The mean and SD for the frequency (percentage) of each cell population within the cohorts, HC (n = 9) and RA (n = 12) are listed with the p-value, the statistical significance and the corrected p-value (Bonferroni).

## Slide 3
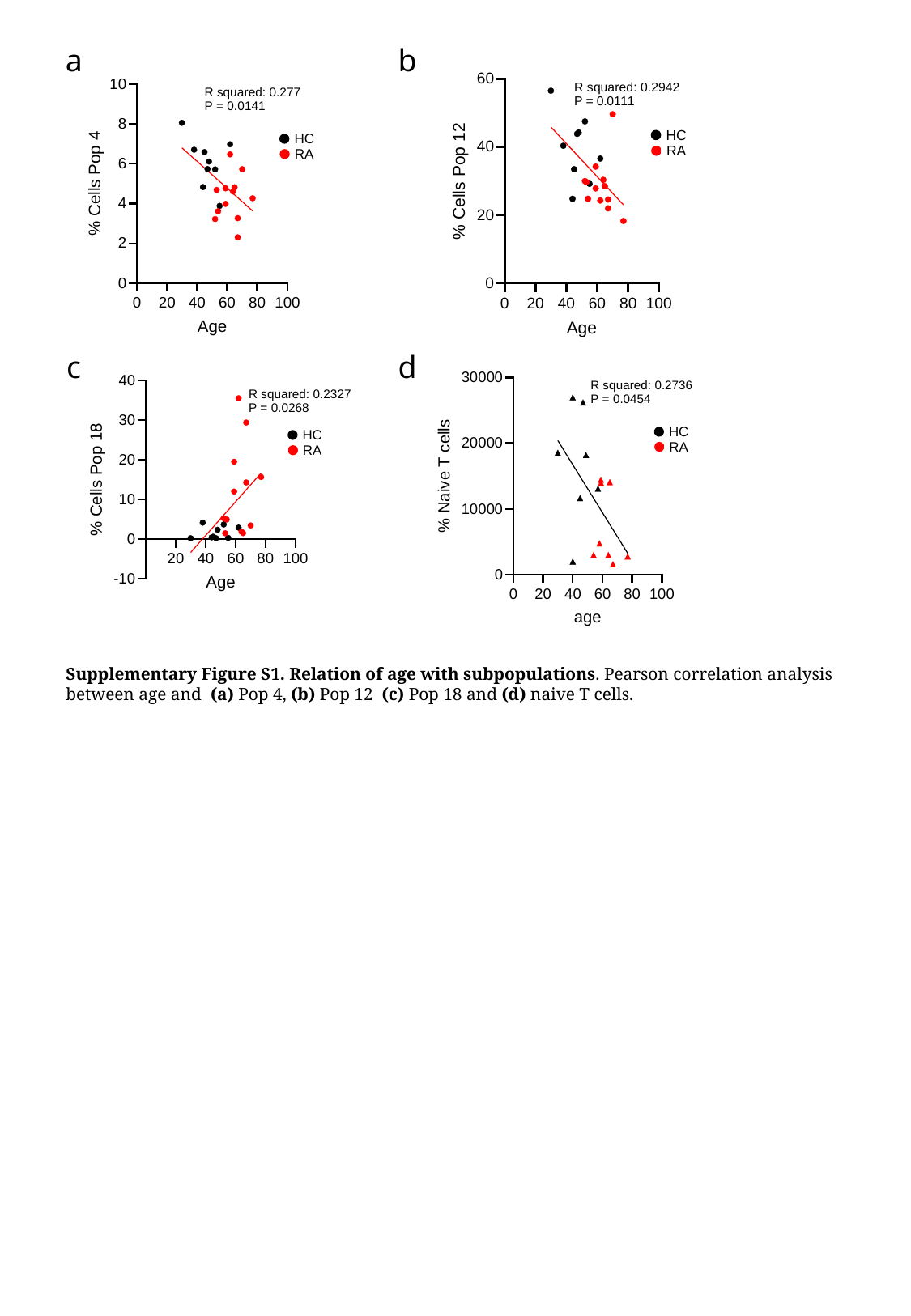

a
b
c
d
Supplementary Figure S1. Relation of age with subpopulations. Pearson correlation analysis between age and (a) Pop 4, (b) Pop 12 (c) Pop 18 and (d) naive T cells.

## Slide 4
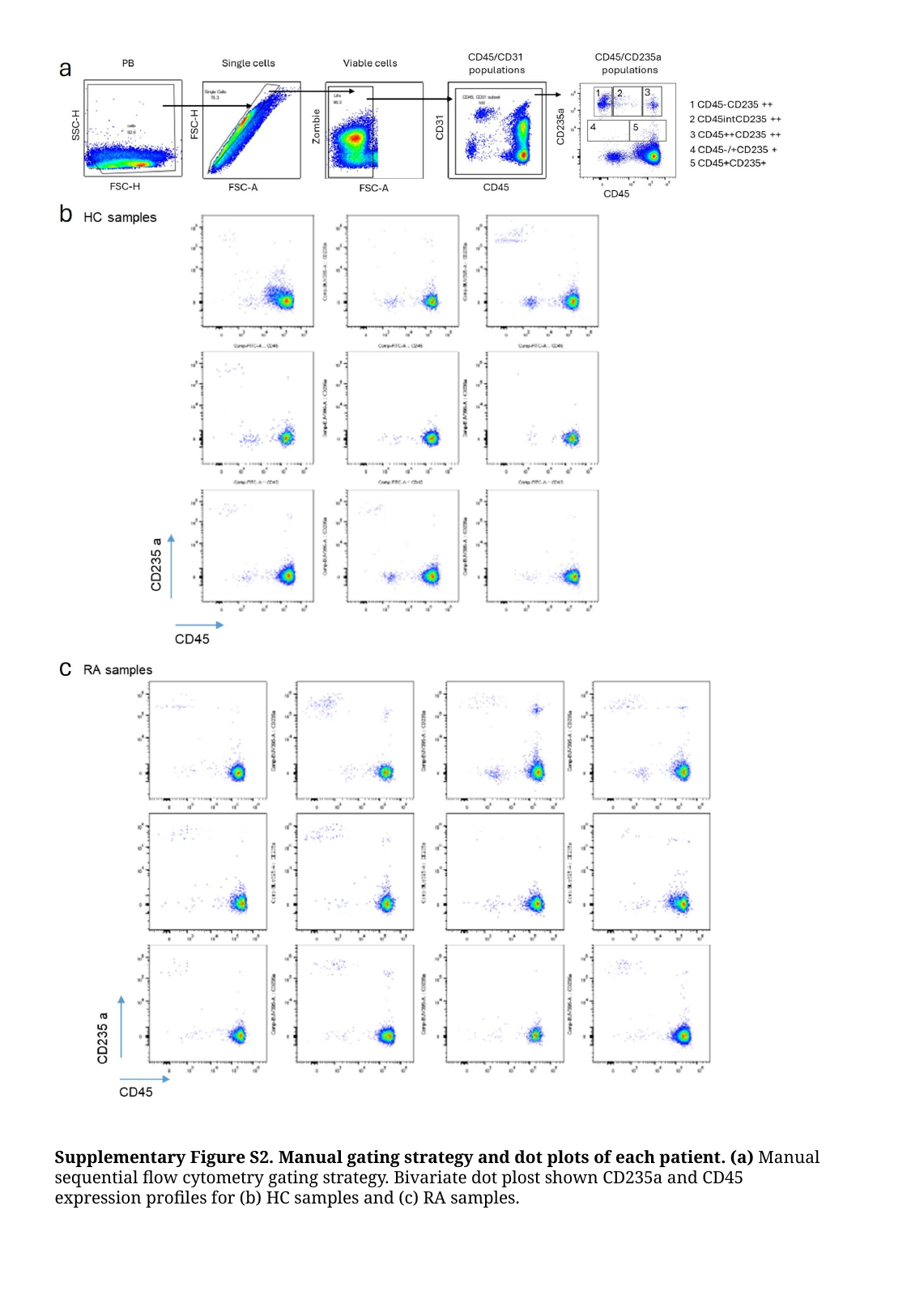

Supplementary Figure S2. Manual gating strategy and dot plots of each patient. (a) Manual sequential flow cytometry gating strategy. Bivariate dot plost shown CD235a and CD45 expression profiles for (b) HC samples and (c) RA samples.
